# The complete mitochondrial genome of *Erromyzon sinensis* (Cypriniformes: Gastromyzontidae) and phylogenetic implications

**DOI:** 10.1080/23802359.2019.1624646

**Published:** 2019-07-11

**Authors:** Lin Wu, Man Zhang, Guangping Cheng, Xiuli Chen

**Affiliations:** a Guangxi Colleges and Universities Key Laboratory of Aquatic Healthy Breeding and Nutrition Regulation, College of Animal Science and Technology, Guangxi University, Nanning, Guangxi, China;; b Guangxi Academy of Fishery Sciences, Nanning, Guangxi, China

**Keywords:** *Erromyzon sinensis*, mitochondrial, genome, phylogenetic tree

## Abstract

In this study, we first determined the complete mitochondrial genome of *Erromyzon sinensis*. It was 16,560 bp in length and contained 13 protein-coding genes, 22 tRNA genes, 2 rRNA genes, a light strand origin of replication (O_L_), and a control region (D-loop). In addition, its overall nucleotide composition was 29.4% of A, 16.8% of G, 25.7% of T, and 28.1% of C, with a total of 55.1% A + T content. Phylogenetic analysis based on 13 protein-coding genes revealed that *E. sinensis* had the closest relationship with *E. kalotaenia*. Our study aimed at providing basic information for molecular systematics and conservation genetics research of *E. sinensis*.

*Erromyzon sinensis*, formerly known as *Protomyzon sinensis*, belongs to the family Gastromyzontidae. It is a native fish which mainly inhabits fast-flowing streams of the West River in Guangxi, southern China (Kottelat 2012). Since 2013, *E. sinensis* was listed as the Data Deficient (DD) on the IUCN red list and the latest red list of Chinese vertebrates (Huckstorf 2013; Jiang et al. 2016). Some researches on the taxonomy of *E. sinensis* have been reported (Chen 1980; Kottelat 2012), but so far there is still no complete mitogenome of this fish. In the present work, we first reported the complete mitochondrial DNA sequence of *E. sinensis*, which will provide basic information for further genetic analysis of this species.

In this study, we collected the specimen of *E. sinensis* from Guilin City (25°16′25.00″N, 110°17′24.07″E) and the muscle sample was preserved in Guangxi Colleges and Universities Key Laboratory of Aquatic Healthy Breeding and Nutrition Regulation, Guangxi University. Genomic DNA was extracted from the muscle sample by using the Qiagen DNeasy Blood and Tissue Kit (Hilden, Germany) according to the manufacturer-recommended specifications. The mitochondrial DNA was amplified through PCR with 14 pairs of primers, then assembled and annotated by using DNAman software (Lynnon Biosoft, Canada) and MitoAnnotator (Iwasaki et al. 2013).

Whole mitochondrial genome sequence of *E. sinensis* had a circular structure of 16,560 bp (GenBank accession no. MH155188), containing 13 protein-coding genes (PCGs), 22 tRNA genes, 2 rRNA (12S and 16S rRNA) genes, a light strand origin of replication (O_L_), and a control region (D-loop), which was displayed a higher conservation than other Gastromyzontidae species. The base composition was 29.4% of A, 16.8% of G, 25.7% of T, 28.1% of C, and A + T-biased (55.1%). All mitochondrial genes were encoded on the H-strand with the exception of eight tRNA genes and one protein-coding gene (*ND6*), which were encoded on the L-strand. Besides, the lengths of 22 tRNAs ranged from 66 bp (*tRNA^Cys^*) to 76 bp (*tRNA^Lys^*). And the *12S* and *16S rRNA* genes were 952 and 1678 bp in length, respectively. The D-loop was located between *tRNA^Pro^* and *tRNA^Phe^*, with a length of 903 bp and a high AT content (63.3%).

To verify the phylogenetic position of *E. sinensis* within Gastromyzontidae, the maximum-likelihood tree was constructed based on 13 PCGs using MEGA 7.0 (Kumar et al. 2016). In addition, two species of the Balitoridae were chosen as outgroups. The GTR + G + I substitution model was selected and recommended by PhyML 3.0 program (Guindon et al. 2010). As shown in the phylogenetic tree, *E. sinensis* had a closer relationship with *E. kalotaenia* than with other species (Figure 1). The results obtained herein would provide reference information regarding family Gastromyzontidae and it would be useful to promote the researches in molecular systematics and conservation genetics of this species.

**Figure 1.**
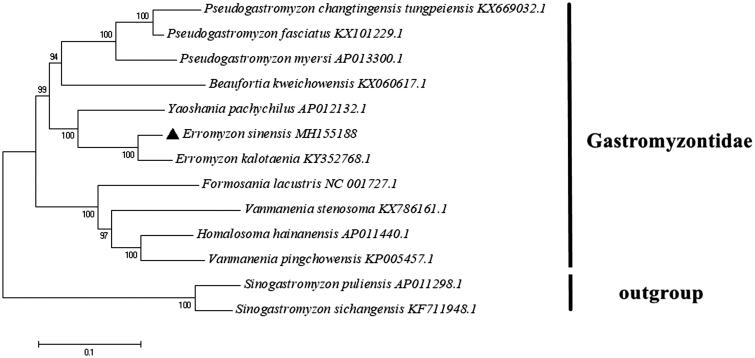
Phylogenetic relationships among *E. sinensis* and 10 other Gastromyzontidae fish based on 13 PCGs. *Sinogastromyzon puliensis* and *S. sichangensis* of Balitoridae were used as outgroups. The phylogenetic tree was performed using MEGA 7.0 software by maximum-likelihood analysis with GTR + G + I model. The GenBank accession number for each species is indicated after the scientific name. The number on branches indicated posterior probabilities in percentage. The mitogenomic information of *E. sinensis* is marked with black triangle.
